# Recent developments in coping strategies focusing on music performance anxiety: a systematic review

**DOI:** 10.3389/fpsyg.2025.1507229

**Published:** 2025-04-25

**Authors:** Parham Bakhtiari, Nazanin Nikanmajd, Ramin Ghasemi Shayan

**Affiliations:** ^1^Department of Classical Music, Music Faculty, Tehran University of Art, Tehran, Iran; ^2^Faculty of Biological Sciences, Islamic Azad University, Isfahan, Iran; ^3^Department of Radiology, Paramedical Faculty, Tabriz University of Medical Sciences, Tabriz, Iran

**Keywords:** anxiety, performance, coping, music, strategy

## Abstract

Music performance anxiety (MPA) is a prevalent concern among musicians, manifesting through cognitive, physiological, and behavioral symptoms that can severely impact performance quality and overall wellbeing. This systematic review synthesizes research on coping strategies employed by musicians to manage MPA from 2016 to 2023, identifying a range of psychological and physical interventions, including Acceptance and Commitment Therapy (ACT), Cognitive Behavioral Therapy (CBT), mindfulness, and yoga. Findings reveal that these interventions significantly reduce anxiety and enhance psychological resilience, with ACT showing notable improvements in psychological flexibility. Physical approaches also proved effective in mitigating physiological symptoms associated with MPA. However, challenges such as small sample sizes and methodological limitations hinder the generalizability of results. The review underscores the necessity for multi-faceted intervention strategies tailored to the unique needs of different musicians, and emphasizes the importance of future research employing larger, randomized controlled designs to further validate these findings. Overall, this review serves as a comprehensive resource for musicians seeking effective coping strategies for managing performance anxiety, highlighting the critical interplay between mental and physical approaches in promoting optimal performance outcomes.

## Introduction

Performance anxiety is a set of disorders that affect people in a series of activities, from test-taking, math performance, public speaking, sport and the performing arts in dance and music (Kenny and Osborne, 2006). Musicians with performance anxiety can suffer from a diversity of cognitive, somatic, and behavioral symptoms of anxiety (Dempsey and Comeau, 2019). On the other hand, coping was demarcated as a musician's behavioral and cognitive efforts to sidestep and diminish the effects of possibly stressful situations (Biasutti, 2014). It refers to individuals' skills to deal with opposing and stressful states. In the musical framework, coping strategies are musicians' behaviors and thoughts to manage music performance anxiety (Burin and Osório, 2017). Previous works suggest that musicians engage in a diversity of coping strategies to moderate MPA, for instance, self-talk, cognitive restructuring, deep breathing, and practicing techniques (Irie et al., 2023). A research has shown that students experiencing adaptive music performance anxiety use a grouping of coping strategies focusing on suitable preparation to preserve a positive attitude to the performance, concentrating on communication with the audience and enjoyment of the music (Sinico da Cunha and Winter, 2013). Music performance anxiety is a noteworthy phenomenon for most music performers, and preceding surveys show a high prevalence of MPA among musicians (Irie et al., 2023). Moreover, recent studies reveal that music performance anxiety is a concern for a substantial majority of undergraduate and professional career musicians (Cruder et al., 2022). Moreover, self-reported anxiety, distress, nervousness, bodily complaints, and negative perceptions are, in most musicians, superior before and during public performances compared to practice (Guyon et al., 2020). Dalila designates that between 60 and 80% of professional musicians suffer from its debilitating forms (Gómez López and Sanchez Cabrero, 2023). In addition, experiencing severe anxiety can be psychologically distressing; over time, chronic anxiety can disturb all aspects of performers' lives, such as wellbeing, identity, self-worth, and relationships (Herman and Clark, 2023).

Music performance anxiety may act as a defense system against traumatic situations, and symptoms may appear in diverse formations. MPA symptoms are typically separated into physiological, cognitive, and behavioral (Moura and Serra, 2021). It is generated in different performance contexts, but it is more intense when the performer is overly worried about their image, there is fear of evaluation and judgment from others, and there is a dread of making mistakes (Moral-Bofill et al., 2022). According to physiological symptoms, one can cite the increased heart rate, heart palpitation, shortness of breath, hyperventilation, dry mouth, sweating, nausea, diarrhea, dizziness and also other physical symptoms, such as headache, digestive difficulties, excessive sweating, musculoskeletal difficulties, muscle tension, cold hands, fatigue, and changes in blood pressure, heart rate, and respiratory rate (Burin and Osório, 2017). Cognitive symptoms can be exhibited as heightened, with a more significant focus on the possibility of making mistakes rather than on the interpretation itself, accelerated tempo due to different time awareness, amplified attention to irrelevant tasks, loss of attention, self-awareness and sense of control or, on the other hand, can be effective with negative thoughts and feelings that could even lead to depression (Moreno-Gutiérrez et al., 2023). Osborne and Franklin (2002) pointed out that the origins of cognitive symptoms have focused on typical cognitive social phobic themes of fear of failure, fear of social disapproval, and irrational and self-defeating thought forms. Some researches were cited regarding behavioral symptoms, like technique failures, posture loss, and tremors (Jucan et al., 2022). Moreover, Juncos et al. (2017) classified behavioral symptoms into two groups, namely overt avoidance behaviors and covert avoidance behaviors. Researchers usually divide anxiety into two types: state anxiety and trait anxiety that are considered a lack of control (Pacheco-Unguetti et al., 2010). State anxiety more closely is similar to fear, while trait anxiety denotes the tendency of an individual to feel chronically worried (Kenny, 2016). These two concepts, are interrelated, therefor, individuals with high-trait anxiety can experience severe anxiety compared to ones with low-trait anxiety (Tovilovic et al., 2009). Since the lack of a study based on the collection of all theoretical studies and practical experiments focusing on the statistical population of musicians and examining their results was felt in the previous researches, in this article, it is attempted that the reader, in addition to familiarizing himself with all aspects of this disorder, by referring to a collection of all the articles done in this field, be aware of the results and the method of conducting experiments. This review begins by recognizing the high prevalence of MPA among professional and student musicians, as highlighted in studies like Kenny (2016). Previous research emphasizes that coping strategies must address both the mental and physical aspects of anxiety to achieve sustainable improvement. While a variety of interventions have been explored, from structured therapies like ACT and CBT to holistic practices such as yoga and mindfulness, their comparative effectiveness remains under-examined. This systematic review aims to bridge that gap by synthesizing evidence on the efficacy of these interventions, identifying recurring themes, and offering tailored recommendations for musicians. In addition, this article aims to express the importance of coping strategies for musicians who encounter music performance anxiety and present a critical systematic review toward the comprehensive evaluation of current coping strategies. In this review, a complete list of coping strategies regarding music performance anxiety has been analyzed to date. This can be a solid reference for musicians in developing music performance and solving the abovementioned issues. The overarching research questions are: “What interventions have been developed in musical societies?” “What are the specific benefits and limitations of these interventions for different groups of musicians?”

## Method

### Search strategy and study selection

Music performance anxiety (MPA) affects musicians across various expertise levels, from amateur performers to seasoned professionals. This systematic review specifically focuses on studies involving professional musicians, music students, and amateur performers. The diversity in participant backgrounds allows for a comprehensive evaluation of how coping strategies are applied in different contexts. The findings aim to address musicians who actively perform, particularly those in educational or professional settings where high performance standards often exacerbate anxiety. By tailoring recommendations to these groups, this article seeks to provide actionable insights for both practitioners and researchers. A comprehensive search was conducted across five electronic databases: PubMed, Scopus, PsycINFO, Web of Science, and Google Scholar. Additionally, we included sources from Sagepub. The search was conducted from 2016 to 2023, targeting studies published in English. We used a combination of keywords related to “Music Performance Anxiety (MPA)” and “coping strategies.” Terms such as “team-based learning,” “stress management,” and “music performance” were employed to ensure comprehensive coverage of relevant studies. A total of 176 records were initially identified through database searches. After removing 51 duplicate records, 129 articles were screened based on the title and abstract for relevance. During the screening phase, 47 studies were excluded for not meeting the established inclusion criteria. Full-text articles of 82 studies were then assessed for eligibility. Articles were excluded for the following reasons: the oldness of the articles (*n* = 17), focus on music as a therapeutic resource rather than performance (*n* = 11), unrelated to musical performance (*n* = 7), review articles or systematic reviews (*n* = 4), studies on non-musical performance (*n* = 2), and articles that did not provide specific coping strategies (*n* = 1). After applying the inclusion and exclusion criteria, 13 studies were deemed eligible and included in the final analysis. The selected studies included participants from diverse musical backgrounds: professional musicians (those engaged in orchestras, ensembles, or solo performances at advanced levels (, music students (Including undergraduate and postgraduate performers studying at conservatories or music school (and Amateur performers (individuals performing for personal or recreational purposes without formal training).

### Inclusion criteria

The articles included in this systematic review were selected based on specific inclusion criteria to ensure relevance and methodological rigor. These criteria were established to focus the review on empirical studies that investigate coping strategies for managing music performance anxiety (MPA). The following key points guided the selection process:

- *Article Availability and Accessibility:* Only freely available and published articles were considered, ensuring that all included studies were verifiable and publicly accessible.
- *Population:* Studies targeting professional musicians, music students, music teachers, and amateur performers were included to capture a broad perspective on MPA management across different expertise levels.
- *Methodological Rigor:* Articles employing structured evaluation methods, such as validated questionnaires, observational studies, face-to-face interviews, or randomized controlled trials (RCTs), were prioritized. This approach ensured that the studies presented reliable and objective data.
- *Outcome Focus:* Only studies measuring the effectiveness of interventions or coping strategies specifically aimed at managing MPA symptoms were included, regardless of participant gender or age.
- *Language:* To maintain consistency and accessibility, only articles published in English were included.

### Exclusion criteria

The approach taken in this paper which displayed in the PRISMA below shows how some articles did not meet inclusion criteria. After removing duplicate articles, some references were from books, chapter of books or dissertations that focused on general aspects of MPA. In addition, some articles were not up-to-date or had a music therapy aspect and did not focus on the music performance features. Four articles were reviews or systematic reviews and one of the articles did not provide a countermeasure. Therefore, only 13 studies were included in the review.

**Figure 1:**
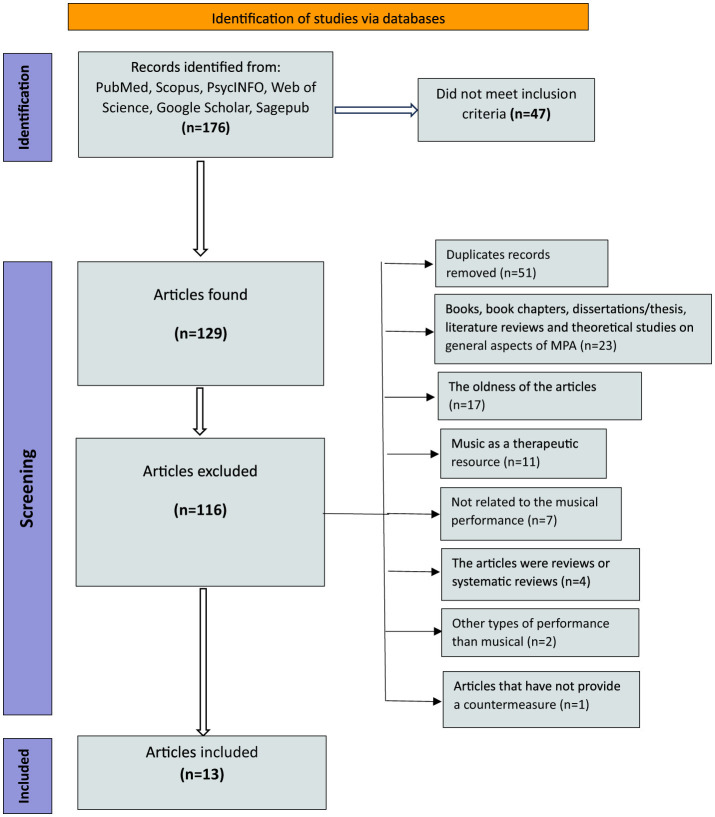
PRISMA flow diagram: a summary of the study selection process for the systematic review.

Of the 13 studies analyzed, (*n* = 2) articles investigated the effect of ACT coping strategy to evaluate the reduction of performance anxiety on vocalists. Moreover, the ACC intervention was tested with the effect of ACT in (*n* = 2) articles to evaluate the effectiveness on symptoms of MPA. The (n=2) articles evaluate the relationship between music performance anxiety and flow experience among professional and non-professional musicians. The effect of yoga and meditation strategy was investigated in (*n* = 1) article by using a control group and an experimental group to evaluate the effect of this method on reducing the amount of MPA. Investigating the simultaneous effect of CH and EMDR methods on an experimental group to reduce MPA and comparing its results with a control group was done in (*n* = 1) article. In (*n* = 1) article, the combination of two CBT and AS strategies on orchestral and private musicians and vocalists examines and determines its relationship with MPA rate. Two articles with the approach of evaluating different coping strategies (mental, physical and performance) adopted by musicians, through different period of times in order to reduce MPA, was examined. One article using mindfulness course and another study tested the effect of EWI (Expressive Writing Intervention) on MPA rate. Figure 1 illustrates the number of records identified, screened, assessed for eligibility, and ultimately included in the analysis. The inclusion and exclusion criteria, along with reasons for exclusions, are visually represented to ensure transparency in the selection of studies.

### Data extraction and synthesis

Data extraction was conducted using a structured template to ensure consistency across all studies. Key information, including study design, intervention type, sample characteristics, and outcome measures, was systematically recorded. This process was carried out independently by two reviewers to minimize bias and enhance reliability.

In cases where discrepancies arose during data extraction, a third reviewer was engaged to resolve conflicts. This resolution process followed a structured protocol:

*i. Identification of discrepancies:* both reviewers highlighted areas of disagreement in their extraction sheets, including differences in interpretation of data or coding of study details.
*ii. Discussion and justification:* the reviewers discussed the identified discrepancies, providing justifications for their interpretations.
*iii. Final arbitration:* the third reviewer evaluated the arguments presented, referring to the original study texts for clarification. Their decision was final and incorporated into the dataset.

The analysis process was designed to identify meaningful patterns and themes from the included studies, ensuring they were representative and robust. Quantitative and qualitative data were analyzed separately to maintain methodological integrity:

- *Quantitative data synthesis:*

Quantitative studies were synthesized using thematic analysis, focusing on commonalities in intervention outcomes. Key variables, such as reduction in MPA symptoms, improvements in psychological flexibility, and enhancement of performance quality, were identified and compared across studies.

- *Qualitative data synthesis:*

For qualitative studies, recurring themes were extracted through an iterative process. Each study was reviewed independently by two reviewers to identify patterns in participants' experiences and reported outcomes. The extracted themes were cross-validated to ensure consistency.

- *Theme validation:*

Themes were considered for inclusion if they met the following criteria:

✓ Reported by multiple studies to establish relevance and reliability.
✓ Supported by substantial data within each study, including participant quotes, observational records, or survey responses.
✓ Aligned with the overarching research questions of the systematic review.

- *Cross-Study Comparison:*

Themes from quantitative and qualitative studies were compared to identify areas of convergence and divergence. For instance, psychological flexibility emerged as a recurring theme across multiple intervention types, reinforcing its importance in managing MPA.

## Results

### Study characteristics

A total of 13 studies met the inclusion criteria and were included in this systematic review. The studies varied in terms of sample size, intervention type, and the specific coping strategies implemented to address MPA. The participants ranged from amateur to professional musicians, with most studies focusing on adult populations. The majority of the included studies utilized cognitive-behavioral interventions, mindfulness-based approaches, or acceptance and commitment therapy (ACT) to alleviate MPA. Some studies also examined physical interventions, such as yoga and relaxation techniques. These approaches can be categorized into two distinct groups:

*I. Specific psychological interventions:*

*Acceptance and commitment therapy (ACT):* this therapy focuses on psychological flexibility, enabling performers to accept anxiety rather than avoid it. For example, Juncos et al. (2017) reported significant reductions in performance anxiety among vocalists after ACT interventions (Juncos et al., 2017).

*Cognitive behavioral therapy (CBT):* a widely used method that emphasizes cognitive restructuring to challenge negative thought patterns. Brooker (2018) demonstrated its efficacy in reducing irrational fears and enhancing performance quality in advanced pianists (Brooker, 2018).

*II. Broad physical and mental wellbeing practices:*

*Yoga and mindfulness:* these approaches primarily address the physiological symptoms of anxiety, such as muscle tension and irregular breathing. Butzer et al. (2016) highlighted how yoga reduced heart rate variability and cortisol levels, while Czajkowski et al. (2022) emphasized mindfulness's role in improving emotional regulation and attentional focus (Butzer et al., 2016; Czajkowski et al., 2022).

### Effectiveness of coping strategies

Quantitative studies predominantly employed interventions such as Cognitive Behavioral Therapy (CBT), Acceptance and Commitment Therapy (ACT), and mindfulness-based approaches. These studies focused on measurable outcomes, including reductions in MPA symptoms, performance quality improvements, and psychological flexibility. For instance:

- Juncos et al. (2017) reported a significant reduction in self-reported anxiety scores and improved performance quality following ACT interventions.
- Butzer et al. (2016) demonstrated the effectiveness of yoga in reducing physiological markers of anxiety, such as heart rate variability and cortisol levels.

The outcomes of quantitative studies consistently highlighted the statistical significance of interventions in managing MPA, with effect sizes ranging from moderate to high in reducing both somatic and cognitive symptoms. Qualitative studies provided rich, narrative insights into the personal experiences of musicians undergoing interventions. These studies explored recurring themes, such as:

- Czajkowski et al. (2022): enhanced emotional regulation through mindfulness.
- Tang and Ryan (2020): improved self-efficacy and resilience among performers engaging in expressive writing interventions.

Recurring themes from qualitative analyses emphasized the transformative nature of interventions, with participants reporting a shift in their perception of anxiety from a hindrance to a manageable aspect of performance. Table 1 provides a detailed overview of the study characteristics, including the sample demographics and interventions used.

**Table 1:** Characterization of the samples and methodological aspects used in the studies assessed in the present review.

**Number**	**References**	**Sample**						**Level**	**Profession**	**Study design**	**Strategy or intervention**	**Intervention schedule**
		**Experimental group**			**Control group**							
		**n**	**Age**	**Edu**	**n**	**Age**	**Edu**					
1	Butzer et al. (2016)	60	Avg: 25.2	NI	43	Avg: 23.6	NI	NI	NI	E inter	Y/Me	2-months in 3 different years
2	Juncos et al. (2017)	7	19–31	CS	_	_	_	B/M	Vocalist	PE (PP)	ACT	12 group sessions in 2 months
3	Kenny and Halls (2018)	68	NI	NI	_	_	_	NI	Orchestral and private musicians and vocalists	PE (PP)	CBT/AS	4 group sessions in 1 day
4	Brooker (2018)	46	18–53	PG/ABRSM	NI	NI	NI	P	Pianist	E inter	CH/EMDR	2 weeks of CH or EMDR
5	Cohen and Bodner (2019)	202	Avg:43.22	NI	_	_	_	P	Classical orchestral musicians	Observational study	Flow	_
6	Shaw et al. (2020)	1	19	U	_	_	_	S	Vocalist	PE (PP)	ACT/ACC	6 one-hour sessions
7	Clarke et al. (2020)	31	19–61	U	_	_	_	S	Vocalist	PE (PP)	ACT	2 h group sessions for 6 weeks
8	Czajkowski et al. (2022)	25	18–38	S	_	_	_	NI	Music student	PE (PP)	Mindfulness	8-week mindfulness course
9	Tang and Ryan (2020)	35	18–61	S	_	_	_	SS	Piano student	PE (PP)	Expressive writing	Performing twice between performances
10	Spahn et al. (2021)	363	Avg:32.8	NI	_	_	_	P/NP	Classical orchestral musicians	Observational study	Flow	_
11	Cornett and Urhan (2021)	270	NI	S/T	_	_	_	NI	Music teacher and student	Case study	Mental/ physical/ performance	_
12	Mahony et al. (2022)	6	18-20	CS	_	_	_	SY	Dance and musical theater	PE (PP)	ACT/ACC	6 one-hour sessions
13	Irie et al. (2023)	38	Avg:20.5	CS	_	_	_	S	Music performer or education	Case study	Mental/ physical/ performance	Before and during performance

ACC, acceptance and commitment coaching; ACT, acceptance and commitment therapy; AVG, average; B/M, bachelor and master; CS, college student; CH/EMDR, cognitive hypnotherapy and eye movement desensitization and reprocessing; E inter, experimental inter-groups; NI, not informed; PE (PP), pre and post intervention; PG/ABRSM, post graduate and exam board of the Royal Schools of Music; P, professional; P/NP, professional and non-professional; S/T, student and teacher; SS, secondary school; S, student; SY, second-year; Y/ME, yoga and meditation; U, university.

### Quality assessment

The included studies scored between 5 and 9 on the Newcastle-Ottawa Scale (NOS), indicating moderate to high quality. However, the quality assessment revealed limitations in the selection of participants and the generalizability of the findings to broader populations of musicians. Studies with lower NOS scores generally had smaller sample sizes or lacked control groups, which may have impacted the robustness of the findings. Studies were rated based on criteria related to the selection of participants, comparability of groups, and the adequacy of outcome measures. Each study received a score, and only studies scoring five or above were included in the final synthesis. Table 2 summarizes the quality assessment of the included studies based on the Newcastle-Ottawa Scale.

**Table 2:** Newcastle-Ottawa Scale (NOS).

**References**	**Selection (max 4 stars)**	**Comparability (max 2 stars)**	**Outcome (max 3 stars)**	**Total score (out of 9)**
Butzer et al. (2016)	⋆Representative of the exposed cohort ⋆Selection of the none exposed cohort ⋆Ascertainment of exposure ⋆Demonstration that outcome of interest was not present at start of study	⋆Study controls for age ⋆Study controls for additional factor (e.g., anxiety history)	⋆Assessment of outcome ⋆Sufficient duration of follow-up ⋆Adequacy of follow-up	9/9
Juncos et al. (2017)	⋆Representative of the exposed cohort ⋆Ascertainment of exposure ⋆Demonstration that outcome of interest was not present at start of study	⋆Study controls for age ⋆Study controls for additional factor (e.g., anxiety history)	⋆Assessment of outcome ⋆Sufficient duration of follow-up ⋆Adequacy of follow-up	8/9
Kenny and Halls (2018)	⋆Representative of the exposed cohort ⋆Ascertainment of exposure ⋆Demonstration that outcome of interest was not present at start of study	The details of the age of the participants are not reported ⋆Study controls for additional factor (e.g., anxiety history)	⋆Assessment of outcome ⋆Sufficient duration of follow-up	6/9
Brooker (2018)	⋆Representative of the exposed cohort ⋆Selection of the none exposed cohort ⋆Ascertainment of exposure ⋆Demonstration that outcome of interest was not present at start of study	⋆Study controls for age ⋆Study controls for additional factor (e.g., anxiety history)	⋆Assessment of outcome ⋆Sufficient duration of follow-up ⋆Adequacy of follow-up	9/9
Cohen and Bodner (2019)	⋆Representative of the exposed cohort ⋆Ascertainment of exposure ⋆Demonstration that outcome of interest was not present at start of study	⋆Study controls for age ⋆Study controls for additional factor (e.g., anxiety history)	⋆Assessment of outcome	6/9
Shaw et al. (2020)	⋆Representative of the exposed cohort ⋆Ascertainment of exposure ⋆Demonstration that outcome of interest was not present at start of study	⋆Study controls for age ⋆Study controls for additional factor (e.g., anxiety history)	⋆Assessment of outcome ⋆Adequacy of follow-up	7/9
Clarke et al. (2020)	⋆Representative of the exposed cohort ⋆Ascertainment of exposure ⋆Demonstration that outcome of interest was not present at start of study	⋆Study controls for age ⋆Study controls for additional factor (e.g., anxiety history)	⋆Assessment of outcome ⋆Sufficient duration of follow-up ⋆Adequacy of follow-up	8/9
Czajkowski et al. (2022)	⋆Representative of the exposed cohort ⋆Ascertainment of exposure ⋆Demonstration that outcome of interest was not present at start of study	⋆Study controls for age ⋆Study controls for additional factor (e.g., anxiety history)	⋆Assessment of outcome ⋆Adequacy of follow-up	7/9
Tang and Ryan (2020)	⋆Representative of the exposed cohort ⋆Ascertainment of exposure ⋆Demonstration that outcome of interest was not present at start of study	⋆Study controls for age ⋆Study controls for additional factor (e.g., anxiety history)	⋆Assessment of outcome	6/9
Spahn et al. (2021)	⋆Representative of the exposed cohort ⋆Ascertainment of exposure	The details of the age of the participants are not reported ⋆Study controls for additional factor (e.g., anxiety history)	⋆Assessment of outcome ⋆Adequacy of follow-up	5/9
Cornett and Urhan (2021)	⋆Representative of the exposed cohort ⋆Ascertainment of exposure	The details of the age of the participants are not reported ⋆Study controls for additional factor (e.g., anxiety history)	⋆Assessment of outcome ⋆Sufficient duration of follow-up	5/9
Mahony et al. (2022)	⋆Representative of the exposed cohort ⋆Ascertainment of exposure ⋆Demonstration that outcome of interest was not present at start of study	⋆Study controls for age ⋆Study controls for additional factor (e.g. anxiety history)	⋆Assessment of outcome ⋆Adequacy of follow-up	7/9
Irie et al. (2023)	⋆Representative of the exposed cohort ⋆Ascertainment of exposure	⋆Study controls for age ⋆Study controls for additional factor (e.g. anxiety history)	⋆Assessment of outcome ⋆Sufficient duration of follow-up	6/9

### Data synthesis

A narrative synthesis was used to summarize the findings of the included studies, given the likely heterogeneity of study designs and interventions. The synthesis focused on the types of coping strategies employed by musicians, the effectiveness of each strategy in reducing music performance anxiety, and the common themes and patterns observed across studies. Due to the expected variation in study designs and outcome measures, a meta-analysis was not conducted. Instead, the review synthesizes results qualitatively to provide a comprehensive understanding of the current coping strategies used for MPA. The selected articles were reviewed according to manage MPA and performance quality. Therefore, the aim of these studies is to focus on the direct relation between MPA and performance quality. According to the Table 3 in addition to reducing MPA, some interventions have other positive effects on self-efficacy, psychological flexibility, and state or trait anxiety.

**Table 3:** Summarizing the findings of included articles.

**References**	**Findings**						**Modality**		
	**Performance quality**	**Psychological flexibility**	**State and trait anxiety**	**Self-efficacy**	**MPA**	**Instrument**	**Cognitive**	**Physiological**	**Behavioral**
Butzer et al. (2016)	Increased	_	_	_	Decreased	(DFS-2) (FFMQ) (POMS) (PAQ)	✓		
Juncos et al. (2017)	Increased	Increased	_	_	Decreased	(AAQ-2) (BAFT) (VLQ) (KMPAI) (ACQ)	✓		
Kenny and Halls (2018)	Increased	_	_	_	Decreased	(KMPAIr) (STAI) (PRIME-MD-PHQ) (ASI)	✓	✓	✓
Brooker (2018)	Increased	_	Decreased	_	Decreased	(STAI Y-1 and Y-2) (SRQ)	✓		
Cohen and Bodner (2019)	Increased	_	_	_	Decreased	(DFS-2) (PAI) (MEC)	✓		
Shaw et al. (2020)	Increased	Increased	_	_	Decreased	(BAFT) (AAQ-2) (PHLMS) (KMPAI)	✓		
Clarke et al. (2020)	Increased	Increased	_	_	Decreased	(K-MPAI) (MPFI) (DASS-21) (MHC-SF)	✓		
Czajkowski et al. (2022)	Increased	_	_	_	Decreased	(FFMQ) (MM)	✓		✓
Tang and Ryan (2020)	Increased	_	_	_	Decreased	(OSPP) (Self report)	✓		✓
Spahn et al. (2021)	Increased	_	_	Increased	Decreased	(PQM) (Flow Short-Scale)	✓		
Cornett and Urhan (2021)	Increased	_	_	_	Decreased	(Questionnaire survey)	✓	✓	✓
Mahony et al. (2022)	Increased	Increased	_	_	Decreased	(PHLMS) (AAQ-2) (BAFT) (KMPAI) (ESS) (SCS)	✓		
Irie et al. (2023)	Increased	_	_	_	Decreased	(Questionnaire survey) (Semi-structured interview)	✓	✓	✓

## Discussion

### Overview of findings

This systematic review synthesizes findings from multiple studies focused on coping strategies for Music Performance Anxiety (MPA). A variety of interventions were evaluated, including Acceptance and Commitment Therapy (ACT), Cognitive Behavioral Therapy (CBT), mindfulness, yoga, expressive writing, and flow state techniques. Across the included studies, several recurring themes were identified through a systematic analysis process. Accordingly, the themes identified and their relevance are mentioned as psychological flexibility which emerged as a key theme across studies utilizing ACT and mindfulness-based interventions. This theme was consistently associated with reduced performance anxiety and improved emotional regulation. Studies such as Juncos et al. (2017) and Shaw et al. (2020) emphasized its role in helping musicians accept anxiety as a natural part of performance, thereby minimizing avoidance behaviors and enhancing overall performance quality (Juncos et al., 2017; Shaw et al., 2020). Moreover, themes of cognitive restructuring and emotional regulation were prominent in studies employing CBT and expressive writing interventions. For instance, Brooker (2018) highlighted the effectiveness of challenging irrational beliefs and fostering positive self-talk, which directly impacted performers' confidence and anxiety management. Physical interventions, such as yoga and relaxation techniques, addressed themes related to somatic symptoms of anxiety. Studies by Butzer et al. (2016) and Czajkowski et al. (2022), demonstrated reductions in muscle tension, heart rate variability, and breathing irregularities, underscoring the importance of incorporating physical components into MPA management. In addition, several studies advocated for combining psychological and physical strategies. This holistic theme was evident in interventions that blended cognitive techniques with somatic practices, such as mindfulness courses incorporating breathing exercises (Czajkowski et al., 2022). The inclusion of themes in this review stems from their prevalence across multiple studies, alignment with research objectives, and demonstrated effectiveness in mitigating Music Performance Anxiety (MPA). Themes were validated through triangulating quantitative data (e.g., reductions in anxiety scores) with qualitative insights (e.g., participant testimonials), ensuring a balanced and multidimensional analysis. Selective citation was employed to maintain focus on interventions directly relevant to the research questions, prioritizing studies with robust methodologies and comprehensive findings. For example, articles were included based on their methodological rigor and alignment with the study's objectives, while interventions with overlapping or redundant findings were represented by the most comprehensive studies to avoid repetition. Psychological interventions such as Acceptance and Commitment Therapy (ACT) and Cognitive Behavioral Therapy (CBT) target the cognitive and emotional roots of MPA, offering structured frameworks to reinterpret anxiety as a manageable challenge. Quantitative studies have consistently demonstrated their efficacy through statistically significant outcomes. For instance, ACT's emphasis on acceptance over avoidance fosters psychological flexibility, reducing state anxiety levels (Juncos et al., 2017). Similarly, CBT's cognitive restructuring techniques help performers manage irrational fears, resulting in improved performance quality (Brooker, 2018). These therapies, while requiring trained professionals, provide targeted, evidence-based solutions for the cognitive dimensions of MPA. Physical interventions, including yoga and mindfulness, focus on somatic symptoms, promoting physiological relaxation and emotional balance. These practices are supported by quantitative evidence, such as biomarkers like heart rate variability and cortisol levels, which underscore their effectiveness in alleviating somatic anxiety (Butzer et al., 2016). In addition, their accessibility makes them practical tools for daily use by performers. Qualitative findings complement this data, highlighting the transformative nature of these interventions in enhancing self-confidence, emotional regulation, and overall wellbeing. For instance, Czajkowski et al. (2022) emphasize mindfulness as a key factor in fostering resilience and adaptive responses to anxiety triggers. By synthesizing findings from both qualitative and quantitative studies, this review demonstrates how different methodologies contribute to a comprehensive understanding of MPA management. Quantitative data offer measurable evidence of efficacy, while qualitative insights provide depth and context, capturing the subjective experiences of musicians. For example, mindfulness practices not only reduce anxiety but also enhance focus and emotional resilience, illustrating their dual psychological and somatic benefits. The differentiated roles of these interventions further underscore the importance of tailored strategies. ACT and CBT require professional expertise to address the cognitive and emotional dimensions of MPA, while yoga and mindfulness offer flexible, practical approaches to manage somatic symptoms. Together, these interventions form a holistic framework for addressing the complex, multifaceted nature of MPA, ensuring adaptability to the individual needs and contexts of performers. The selective use of rigorous, comprehensive studies further enhances the reliability and relevance of these findings, ensuring the review's conclusions are well-supported and focused.

### Comparative analysis of interventions

Several intervention modalities emerged as particularly effective. For instance, ACT was consistently reported to improve performers' acceptance of anxiety and reduce avoidance behaviors, leading to enhanced performance quality (Juncos et al., 2017; Shaw et al., 2020). This was reflected across multiple studies where psychological flexibility was a significant mediator in reducing performance anxiety. On the other hand, mindfulness-based interventions demonstrated improvements in both cognitive and physiological measures of MPA, supporting their role in reducing performance-related stress and anxiety (Czajkowski et al., 2022). CBT and hypnotherapy also showed promising results, particularly in studies by Brooker (2018), where cognitive restructuring techniques were used to challenge negative beliefs about performance. Eye Movement Desensitization and Reprocessing (EMDR) was notably effective for advanced pianists, indicating that combining traditional cognitive interventions with newer therapeutic techniques can offer tailored solutions for different levels of performers.

However, the effectiveness of these interventions varies depending on factors such as the duration of the intervention and the level of performer engagement. For instance, Kenny and Halls (2018) reported that shorter interventions might not yield long-term benefits, suggesting that continuous practice and long-term engagement are necessary to fully internalize these coping mechanisms.

### Challenges and limitations

Despite the overall effectiveness of interventions aimed at reducing Music Performance Anxiety (MPA), several challenges emerged across the studies. A common issue was small sample sizes, as seen in studies by Shaw et al. (2020), Mahony et al. (2022), and Czajkowski et al. (2022), which limited the generalizability of findings to broader populations of musicians. Similarly, the lack of control groups in some research, such as Cornett and Urhan (2021), hindered the ability to attribute improvements solely to the interventions. These methodological limitations highlight the need for more rigorous study designs in future research. Another significant challenge was the diversity in the types of coping strategies and interventions examined. The heterogeneity in intervention duration, delivery methods (e.g., group vs. individual sessions), and focus areas created inconsistencies in results and made direct comparisons challenging. For example, while Acceptance and Commitment Therapy (ACT) consistently improved psychological flexibility, interventions like expressive writing (Tang and Ryan, 2020) emphasized cognitive and emotional expression, making it difficult to evaluate outcomes using a unified framework (Tang and Ryan, 2020). The lack of long-term follow-up in many studies was another limitation, as seen in research by Kenny and Halls (2018) and Kenny and Halls (2018). Although short-term effectiveness was often demonstrated, the sustainability of these coping strategies over extended periods remains unclear. This issue was particularly evident in studies that employed short-term interventions, where the long-term effectiveness of the methods could not be assessed. Future research should address these challenges by employing larger, more diverse samples, incorporating control groups, and conducting longitudinal follow-ups to evaluate the persistence of intervention effects over time. Standardizing intervention delivery and measuring outcomes using consistent frameworks would also enhance comparability and the overall reliability of findings.

### Implications for future research

The findings from this review highlight the importance of multi-faceted approaches in addressing MPA. While psychological interventions such as ACT, CBT, and mindfulness have shown considerable promise, future research should explore combining these approaches with physical and performance-based techniques to maximize their effectiveness. Additionally, more attention should be given to the development of interventions tailored to specific types of performers (e.g., instrumentalists vs. vocalists) and their unique challenges in managing performance anxiety. Moreover, future studies should aim to include larger, more diverse populations and use randomized controlled designs to strengthen the evidence base for these interventions. The inclusion of physiological measures, such as heart rate variability and cortisol levels, alongside psychological assessments could offer a more comprehensive understanding of how these interventions impact both the mind and body.

## Conclusion

This systematic review provides an in-depth evaluation of coping strategies for Music Performance Anxiety (MPA), synthesizing findings from quantitative and qualitative studies to highlight the efficacy of diverse interventions, including Acceptance and Commitment Therapy (ACT), Cognitive Behavioral Therapy (CBT), mindfulness, yoga, and expressive writing. The evidence underscores significant reductions in MPA symptoms, with measurable improvements in psychological flexibility, emotional regulation, and performance quality. Additionally, qualitative insights reveal the transformative and personal impacts of these strategies, emphasizing themes of self-efficacy, resilience, and adaptive coping. Despite these promising findings, the review identifies critical gaps in the existing literature that warrant further exploration. Future research should focus on addressing challenges such as small sample sizes, variability in intervention delivery, and limited long-term follow-up. Tailored interventions are needed to accommodate the diverse contexts, skill levels, and anxiety triggers of different groups, such as instrumentalists, vocalists, and ensemble performers. For example, professional orchestral musicians may benefit from strategies addressing performance perfectionism, while amateur performers may require interventions emphasizing emotional resilience. Longitudinal studies are essential to evaluate the sustainability of these strategies and their long-term impact on musicians' careers and overall wellbeing. Furthermore, interdisciplinary approaches integrating psychology, neuroscience, and musicology could foster the development of innovative solutions, such as neurofeedback training or technology-assisted mindfulness. Greater attention should also be given to underrepresented populations, including musicians from non-Western cultures and those with disabilities, to ensure inclusivity in intervention strategies. Overall, this review underscores the importance of integrating evidence-based interventions into musicians' training programs, fostering both mental and physical resilience. By addressing research gaps and enhancing practical applications, these strategies can not only improve performance outcomes but also promote the wellbeing of musicians across all levels of expertise. Bridging the gaps in research and practice will pave the way for more comprehensive, inclusive, and sustainable approaches to managing MPA.

## Data Availability

The original contributions presented in the study are included in the article/supplementary material, further inquiries can be directed to the corresponding author.
